# Oxidative Stress and Male Infertility: The Protective Role of Antioxidants

**DOI:** 10.3390/medicina59101769

**Published:** 2023-10-04

**Authors:** Aris Kaltsas

**Affiliations:** Department of Urology, Faculty of Medicine, School of Health Sciences, University of Ioannina, 45110 Ioannina, Greece; a.kaltsas@uoi.gr; Tel.: +30-265-109-9664

**Keywords:** oxidative stress, male infertility, antioxidants

## Abstract

Oxidative stress is a significant factor in male infertility, compromising sperm function and overall reproductive health. As male infertility garners increasing attention, effective therapeutic interventions become paramount. This review investigates the therapeutic role of antioxidants in addressing male infertility. A detailed examination was conducted on antioxidants such as vitamin C, E, B12, D, coenzyme Q10, zinc, folic acid, selenium, l-carnitine, l-arginine, inositols, and alpha-lipoic acid. This analysis examines the methodologies, outcomes, and constraints of current clinical studies. Antioxidants show notable potential in counteracting the negative effects of oxidative stress on sperm. Based on the evidence, these antioxidants, individually or synergistically, can enhance sperm health and reproductive outcomes. However, certain limitations in the studies call for careful interpretation. Antioxidants are integral in tackling male infertility attributed to oxidative stress. The current findings underscore their therapeutic value, yet there’s a pressing need for deeper, comprehensive research. Future studies should focus on refining dosage guidelines, identifying potential side effects, and discerning the most efficacious antioxidant combinations for male infertility solutions.

## 1. Introduction

Male infertility is a global health concern, affecting 40–50% of couples worldwide [1]. Oxidative stress, one of the primary contributors to male infertility, refers to an imbalance between production of reactive oxygen species (ROS) and antioxidant defense mechanisms of the body. ROS, such as superoxide anions, hydrogen peroxide, and hydroxyl radicals, are formed as by-products of normal cellular metabolism, especially during the mitochondrial electron transport process [2]. However, when produced in excess, especially under conditions of environmental stress, infections, or exposure to toxins, they can cause significant cellular damage [3]. ROS can directly damage the sperm plasma membrane, leading to changes in its fluidity and integrity. This results in compromised sperm functionality, including reduced motility and viability [4]. Studies show that 30–80% of infertile men exhibit elevated ROS levels, which attack and damage deoxyribonucleic acid (DNA) and the proteins and lipids of sperm [5], leading to compromised functionality, including interference with capacitation processes needed for successful fertilization [6,7].

Antioxidants have the ability to mitigate oxidative damage via direct interaction with free radicals, as well as indirectly by decreasing the activity or expression of enzymes that generate free radicals or by improving the activity or expression of intracellular antioxidant enzymes [8]. They present a promising avenue for addressing male infertility treatment challenges, with recent scientific discussions showing their utility in treating this reproductive health condition [9]. While antioxidant supplementation is championed as an effective strategy, rigorous research is essential to establish standard regimens and gauge its efficacy in real-world settings [10].

This review provides a comprehensive examination of the interactions between antioxidants and male infertility, including an in-depth exploration of oxidative stress in male infertility, methods for measuring semen oxidative stress levels and its impact on male reproductive health, antioxidant therapy benefits as a remedy and any possible drawbacks or concerns with using natural antioxidants and phytochemicals in treating male infertility.

The structure of our article has been developed with clarity and depth in mind. To start off our narrative, we will explore the factors contributing to male infertility caused by oxidative stress, with particular focus on ROS-related adverse outcomes. This will then lead to methods of assessing oxidative stress in semen using both standard and advanced techniques, followed by an overview of evidence supporting antioxidant therapy as a potential treatment strategy for male infertility, including both supplements and natural derivatives. Additionally, we will explore the use of oxidation–reduction potential (ORP) as a marker of male infertility, while discussing genetic variations affecting antioxidant genes as potential contributors. Finally, our concluding sections will highlight clinical relevance of oxidative stress assessments and practical applications of antioxidants in managing male infertility.

This study aims to highlight the nuances of antioxidants as therapeutic avenues for male infertility. Our findings aim to augment the current understanding, informing clinical practices about the potential of antioxidants as treatment solutions for male infertility.

## 2. Oxidative Stress and Male Infertility

Oxidative stress occurs when the production of free radicals, like ROS, surpasses the body’s ability to neutralize them with antioxidants. This imbalance can result in cellular damage. Additionally, it disrupts essential biological processes. A significant area affected by oxidative stress is male reproductive health. Thus, oxidative stress is crucial when exploring male infertility [11].

### 2.1. Dual Functionality of ROS

**Figure 1:**
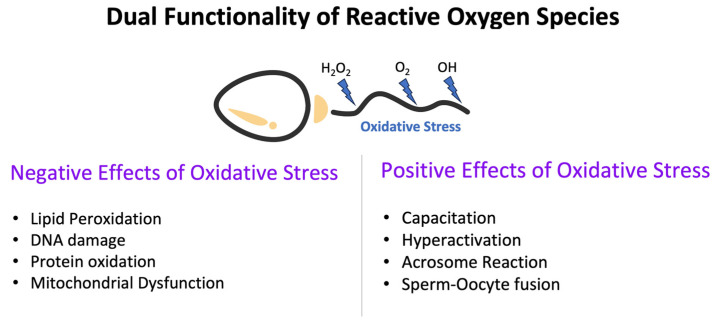
Dual role of oxidative stress in male reproduction.

ROS are chemically reactive molecules containing oxygen. These molecules, mainly produced as metabolic by-products during mitochondrial respiration and various enzymatic reactions, have both positive and negative effects on male reproduction [12]. Under the controlled and balanced conditions, ROS are beneficial, fostering essential physiological processes like sperm capacitation, acrosome reaction, and intercellular signaling [13]. However, an excessive accumulation of ROS becomes pathological, instigating a sequence of cellular damages detrimental to sperm function [14]. For a visual representation of the dual role of oxidative stress in male reproduction, refer to Figure 1 below.

### 2.2. Major Producers of ROS in the Reproductive System

To understand the complex mechanisms of oxidative stress, one must first understand its primary actors. Spermatozoa, or sperm cells, are pivotal in the ROS landscape. As they mature and capacitate, there is a surge in ROS generation, steered by the activation of enzymes such as nicotinamide adenine dinucleotide phosphate (NADPH) oxidase [15,16]. This ROS formation is a component of capacitation and hyperactivation, both of which are vital for sperm function and the fertilization process. However, an excessive amount of ROS produced by sperm cells can cause oxidative damage to their membranes, DNA, and other cellular components, leading to decreased sperm motility, reduced viability, and a diminished potential for fertilization [16,17].

Leukocytes, which are white blood cells, further intensify the ROS production, particularly when releasing inflammatory substances [18]. These immune cells found within the male reproductive tract can generate ROS to defend against infections. When activated by inflammatory processes or infections, leukocytes produce ROS as a defense mechanism against pathogens. However, in situations like persistent infections or inflammation, the ROS production by leukocytes can become excessive, leading to oxidative stress. This can adversely affect sperm function and contribute to male infertility [19].

Moreover, adjacent tissues, such as the prostate gland and seminal vesicles, also produce ROS through enzymatic reactions. Seminal plasma, the fluid that nourishes and surrounds sperm cells, contains a variety of enzymes and chemicals capable of producing ROS. These include enzymatic components like NADPH oxidase and xanthine oxidase, as well as molecular constituents such as prostaglandins and lipid peroxides. Their presence in seminal plasma contributes to the oxidative environment within the male reproductive system. While ROS generation in seminal plasma serves important physiological roles, an overproduction can overwhelm the antioxidant defenses, leading to oxidative stress that negatively impacts sperm quality and fertility [15].

However, these endogenous sources are merely part of the overall picture. The modern world, with its trove of environmental pollutants, radiation from electronic devices, and stressful lifestyles, has become an inadvertent contributor to male oxidative stress. Understanding the sources of ROS generation within the male reproductive system is essential to comprehend the processes of oxidative stress and its effects on male fertility. To mitigate the negative effects of oxidative stress on male reproductive function and improve fertility outcomes, it is imperative to balance ROS production with antioxidant defense systems [20].

### 2.3. Effects of Oxidative Imbalance on the Reproductive System

When ROS overproduction remains unchecked, the cascade of ensuing damages is extensive:

Lipid Peroxidation: The sperm membrane, rich in polyunsaturated fatty acids, is particularly susceptible [21]. ROS target the polyunsaturated fatty acids present in the membranes of sperm cells, triggering a cascade of events known as lipid peroxidation. This biological mechanism leads to the generation of lipid peroxides and the perturbation of cellular membrane integrity. Consequently, the functionality and survivability of sperm are affected, resulting in decreased motility and diminished fertilization potential [21].

DNA Fragmentation: One of the most poignant implications of oxidative stress is its potential to fragment DNA and instigate oxidative modifications. This not only challenges the genetic integrity of sperm but also casts a shadow on its fertilizing potential [22]. Oxidative stress can lead to DNA strand breaks, impacting sperm’s fertilization capacity. Such fragmentation is linked to embryo abnormalities, impacting its development and increasing the chances of early pregnancy loss [23]. In addition to fragmentation, oxidative stress can cause genetic mutations and chromosomal abnormalities in sperm DNA. These anomalies can lead to developmental defects in embryos and a heightened miscarriage risk [24]. The DNA in sperm cells, due to its high degree of compaction and tight packaging, is especially vulnerable to oxidative damage from ROS. This damage has been linked to reduced fertilization rates, compromised embryo quality, and an increased likelihood of developmental defects in offspring [22].

Protein Oxidation: ROS can induce oxidative modifications in proteins present in spermatozoa, leading to changes in their structural integrity and subsequent degradation of their functional capabilities. The proteins may undergo oxidative damage, affecting various aspects of sperm physiology, such as motility, DNA packing, and fertilization capability [25].

Mitochondrial Dysfunction: Mitochondria play a central role in energy production through oxidative phosphorylation, generating the adenosine triphosphate (ATP) essential for sperm motility and vitality [26]. Given the pivotal role of mitochondria in energy production, their damage can significantly reduce sperm motility and vitality, diminishing reproductive potential [27]. When exposed to oxidative stress, mitochondria can undergo damage, leading to impaired function and reduced ATP production. This impairment can significantly diminish sperm motility and vitality. Oxidative stress directly affects mitochondrial function by damaging its membranes and impairing the electron transport chain (ETC) complexes. ROS can induce lipid peroxidation in mitochondrial membranes, altering membrane fluidity and disrupting the ETC. Such disruptions result in decreased ATP production and compromised mitochondrial function [28]. Moreover, ROS can directly target ETC complexes, especially complex I and complex III, causing electron leakage and amplifying ROS production, which further intensifies oxidative stress and mitochondrial dysfunction [26]. ATP, produced by mitochondria, is vital for maintaining sperm motility as it fuels flagellar movement. Any reduction in ATP production due to impaired mitochondrial function can lead to decreased sperm motility, which in turn affects fertility [26]. Research has underscored the strong correlation between mitochondrial enzyme activities and sperm motility, emphasizing the reliance of motility on optimal mitochondrial function [26]. Furthermore, mitochondrial dysfunction can also impact sperm vitality. Mitochondria are instrumental in regulating cellular apoptosis. When their function is compromised, this regulation can be disrupted. Oxidative stress-induced mitochondrial dysfunction can escalate apoptotic signaling and programmed cell death in sperm, diminishing their vitality and viability [29]. Such a scenario can lead to a reduced sperm count and overall reproductive potential.

These mechanisms together highlight the profound influence of oxidative stress on male reproductive function. The processes of lipid peroxidation in sperm cell membranes, combined with protein oxidation and DNA damage, contribute to the decline in sperm motility, reduced reproductive capacity, and increased susceptibility to infertility. Understanding these pathways is crucial to elucidating the role of oxidative stress in male infertility. The importance of implementing strategies to reduce oxidative damage, such as the use of antioxidant supplements, is emphasized to protect and enhance male reproductive health [14,30].

### 2.4. Wider Implications of Oxidative Stress on Sperm Health and Reproductive Potential

#### 2.4.1. Effects of Oxidative Stress on Sperm Parameters

Sperm Count: Oxidative stress, primarily due to elevated ROS levels, can impair sperm viability and reduce sperm count [31]. ROS can induce cell death pathways in sperm, leading to decreased sperm viability and increased apoptosis [32]. Oxidative stress can also affect sperm production in the testes, leading to reduced sperm count and oligozoospermia [33]. Furthermore, oxidative stress can disrupt the antioxidant defense system in the seminal plasma, leading to decreased antioxidant capacity and increased susceptibility to oxidative damage [34].

Sperm Motility: The oxidative stress hampers the sperm’s ability to move effectively. By hampering the flagellar movement, ROS hinder the forward progression of sperm, culminating in asthenospermia, which negatively impacts fertilization potential [35]. ROS can directly affect the sperm plasma membrane, leading to changes in membrane fluidity and integrity. This can result in reduced sperm motility and impaired forward progression, making it more difficult for sperm to reach and fertilize the oocyte [23]. Additionally, oxidative stress can disrupt the normal processes of sperm capacitation, which is necessary for sperm to acquire fertilization competence. Capacitation involves changes in sperm membrane fluidity, ion channel activity, and protein phosphorylation. ROS can interfere with these processes, leading to impaired capacitation and reduced sperm motility [36].

Sperm Morphology: ROS-induced oxidative stress can lead to direct damage to the sperm plasma membrane, causing changes in its fluidity and integrity [37]. This results in structural deformities in sperm, known as teratospermia, which compromise successful fertilization and decrease the likelihood of a healthy conception [38]. Such alterations in the shape and structure of the sperm are further exacerbated by ROS damage to the mitochondria, crucial for sperm function and energy production [37]. Mitochondrial dysfunction can lead to structural abnormalities in the sperm, affecting its morphology. Furthermore, ROS can induce DNA damage in sperm, which can also contribute to structural deformities. Oxidative stress can lead to DNA strand breaks, base modifications, and cross-linking, resulting in genetic abnormalities and chromosomal aberrations [39]. These DNA lesions can affect the proper packaging of DNA within the sperm nucleus, leading to abnormalities in sperm morphology [40]. The integrity and stability of the sperm DNA are essential for normal sperm development and function. Moreover, ROS can disrupt the normal processes of sperm maturation and capacitation. Capacitation is a series of biochemical and physiological changes that sperm undergo in the female reproductive tract to acquire fertilization competence. ROS can interfere with these processes, leading to impaired sperm function and morphology [41]. ROS can also affect the acrosome reaction, which is necessary for sperm penetration of the egg during fertilization [42]. The disruption of these critical processes can result in structural deformities in sperm. Additionally, oxidative stress can affect the cytoskeletal components of sperm, such as microtubules and microfilaments, which are essential for maintaining sperm morphology and motility [36]. ROS can disrupt the organization and stability of these cytoskeletal structures, leading to structural abnormalities in sperm.

Sperm Function: The physiological functions of sperm are crucial for successful fertilization. Controlled levels of ROS are essential for several physiological processes in male reproductive function, including sperm capacitation, hyperactivation, acrosome response, and sperm–oocyte fusion. The process of spermatozoal maturation, which occurs in the epididymis, involves changes in the cell membrane, the reorganization of surface proteins, and nuclear and enzymatic modifications [35]. ROS plays a pivotal role in these processes, facilitating the formation of disulfide bonds, contributing to chromatin stability, and protecting DNA integrity [43]. ROS also serves as signal transducers in many physiological stages of sperm function, including maturation, activation, capacitation, and acrosome response [44].

Capacitation: Capacitation is the final functional step in sperm development, making them capable of fertilizing an ovum. ROS promotes capacitation by activating intracellular cyclic adenosine monophosphate (cAMP) levels, leading to the subsequent activation of protein kinase A (PKA) [45,46].

Hyperactivation: This refers to a specific condition where sperm motility exhibits distinct characteristics, essential for the effective penetration of the zona pellucida by sperm during fertilization [47]. ROS has been shown to positively influence hyperactivation mechanisms in sperm [48].

Acrosome Response: For successful fertilization, sperm must traverse the cumulus oophorous, attach to the zona pellucida surrounding the egg, and initiate the exocytotic release of proteolytic enzymes [43]. ROS plays a role in facilitating activities on the zona pellucida of the sperm, achieved via the phosphorylation of specific plasma membrane proteins [44].

Sperm–Oocyte Fusion: ROS enhances the cell membrane’s fluidity, which is essential for the effective fusion of sperm and oocyte. This is achieved by facilitating the biochemical processes of sperm capacitation and acrosome responses [49].

In conclusion, while ROS can have detrimental effects on sperm health, controlled levels play a crucial role in various physiological processes essential for successful fertilization. Proper understanding and management of ROS levels are pivotal for male reproductive health.

**Figure 2:**
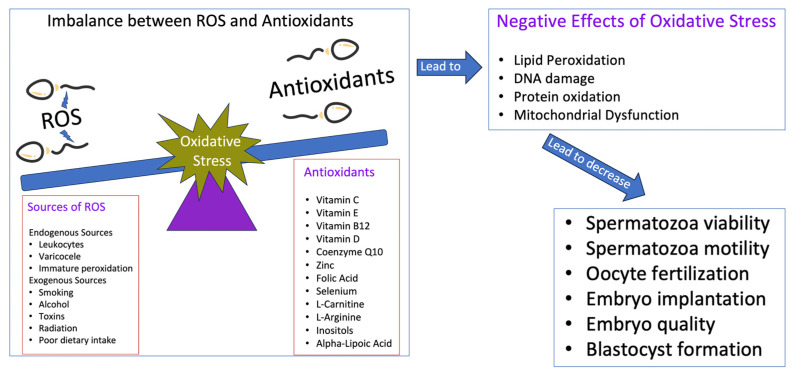
Imbalance between ROS levels and antioxidants: implications for sperm quality and male reproductive health.

The intricate relationship between ROS levels, antioxidants, and their collective impact on sperm quality and male reproductive health is multifaceted. Figure 2 provides a comprehensive illustration of the delicate balance between ROS-induced oxidative stress and the protective mechanisms of antioxidants, highlighting their implications for sperm quality and the broader spectrum of male reproductive health.

#### 2.4.2. Effects of Oxidative Stress on Reproductive Potential

Oxidative stress has repercussions that extend beyond mere fertility concerns:

Impact on male reproduction leading to recurrent miscarriages in females and genetic anomalies in offspring: Elevated oxidative stress in males can have repercussions on female fertility [24]. Specifically, increased oxidative stress in sperm can correlate with a higher incidence of miscarriages in females and heightened risk of genetic disorders in offspring [50]. This underscores the importance of understanding the broader implications of male oxidative stress on overall reproductive outcomes.

Childhood Ailments: Offspring conceived via assisted reproductive techniques might be predisposed to certain childhood diseases due to the oxidative stress in the paternal system [51,52].

Hormonal Imbalances: The multifaceted influence of oxidative stress extends to hormonal regulation, potentially creating broader reproductive health challenges [15].

In conclusion, the delicate balance between ROS and antioxidants in the male reproductive is pivotal not only for sperm health but also overall male reproductive well-being and the health of future offspring. As our comprehension of this relationship deepens, it underscores the importance of exploring both diagnostic and therapeutic strategies to address oxidative stress, aiming for enhanced reproductive outcomes.

## 3. Antioxidants and Reproductive Health

Antioxidants, widely recognized for their role in combating cellular damage, have emerged as pivotal players in maintaining and enhancing male reproductive health. Their ability to counteract the damaging effects of reactive oxygen species makes them an essential component in safeguarding sperm function and integrity. Antioxidants are compounds proficient in mitigating the adverse impacts of ROS, acting as safeguards against oxidative stress. They fall into two primary categories:

1. Enzymatic Antioxidants: These include enzymes like superoxide dismutase (SOD), catalase, and glutathione peroxidase (GPx) and are central to combating oxidative stress by breaking down harmful ROS into harmless components [53].
2. Nonenzymatic Antioxidants: This broad group encompasses vitamins, minerals, carotenoids, and flavonoids. Notable examples include vitamins C and E, which neutralize free radicals, and minerals like selenium and zinc that support enzymatic antioxidants. Dietary carotenoids and flavonoids further fortify the body’s antioxidant defenses [54].

Together, these antioxidants provide a robust defense against oxidative stress, enhancing sperm health and male reproductive function.

Dietary Sources of Antioxidants

- Fruits and Vegetables: Berries offer anthocyanins, citrus fruits are a source of vitamin C, and vegetables such as tomatoes and spinach deliver a blend of vitamins and other antioxidant compounds [55].
- Nuts and Seeds: Sources of essential nutrients, including antioxidants. Examples include almonds with vitamin E and flaxseeds rich in lignans [56].
- Whole Grains: Besides fiber, grains like brown rice and quinoa offer antioxidants such as selenium [57].
- Legumes: Beans and lentils, rich in flavonoids, contribute antioxidants alongside their other nutritional benefits [58].
- Herbs and Spices: Flavor enhancers like turmeric, cinnamon, and garlic are also potent sources of antioxidants [59].

In conclusion, antioxidants play an instrumental role in promoting male reproductive health, ensuring sperm integrity and optimizing fertility potential. Incorporating antioxidant-rich foods into one’s diet can further support this cause.

## 4. Antioxidants Supplements and Male Fertility Enhancement

### 4.1. Role of Antioxidant Supplementation in Male Reproduction

Antioxidant supplements introduce external antioxidants into the body, individually or in combinations, aiming to bolster its natural defense against oxidative stress. By attempting to balance ROS and antioxidants, this supplementation has potential benefits for male reproductive health [60].

#### 4.1.1. Positive Effects on Sperm Health

Research has consistently indicated the positive influence of antioxidant supplements on sperm attributes. Specifically, they have been linked with:

DNA Fragmentation Reduction: Antioxidants are crucial in safeguarding sperm DNA from oxidative damage. Spermatozoa, when exposed to ROS, can experience DNA fragmentation and chromosomal abnormalities [61]. However, antioxidants not only prevent this DNA damage by curbing ROS formation and reducing oxidative stress [62] but also mend lesions on the DNA molecules, ensuring the preservation of the genetic material’s integrity [24]. Furthermore, alongside the sperm solution’s pH, antioxidants can significantly enhance the fertilization capabilities of sperm. Without these protective measures, the sperm’s ability to maintain DNA can be compromised, leading to diminished fertilization potential [63].

Sperm Count and Quality: Antioxidants play a pivotal role in enhancing sperm quality by increasing count, motility, viability, and improving morphology. Oxidative stress can impair sperm function, leading to reduced fertility [62]. By curbing the damage from oxidative stress on sperm cells [64,65,66,67], antioxidants not only protect their structural and functional integrity [68] but also bolster motility through the maintenance of membrane fluidity and stimulation of energy production [69]. Furthermore, they safeguard against DNA fragmentation and morphological abnormalities [70]. Antioxidants modulate the levels of oxidative stress and ROS in seminal plasma. Seminal plasma contains antioxidant mechanisms, which can quench ROS production and protect spermatozoa against potential damage [71]. By lowering ROS levels, antioxidants create an ideal environment for sperm function and fertility.

#### 4.1.2. Fertility Outcomes and Antioxidant Supplementation

Coupled with sperm quality enhancement, antioxidant supplements have also been connected with promising fertility results. For couples aspiring to conceive:

Higher Conception Rates: Antioxidants play a crucial role in enhancing fertility potential by improving reproductive outcomes. Their usage by the male partner correlates with more successful pregnancy outcomes and higher rates of live births, emphasizing their significance in creating an environment favorable for fertilization, embryo growth, and subsequently, successful pregnancies [72]. In vitro fertilization (IVF), a widely used assisted reproductive technology, has seen improved outcomes with antioxidant supplementation, including better fertilization rates, embryo quality, and motility [62]. These compounds protect sperm cells during cryopreservation, reducing cryodamage and preserving quality [68]. In the realm of animal husbandry, antioxidants have boosted male poultry fertility by elevating live sperm count, enhancing the total antioxidant capacity of seminal plasma, and ensuring membrane integrity [69]. On a molecular level, antioxidants are instrumental in regulating DNA damage response (DDR) pathways and repair mechanisms. The DDR is a sophisticated network of signaling pathways that detect and rectify DNA damage [73]. Antioxidants can activate DDR pathways to facilitate the repair of oxidative DNA damage, maintaining genome integrity. They also modulate DNA repair pathways, adjusting their activation in response to oxidative stress, ensuring efficient DNA damage repair and minimizing mutation accumulation [73].

### 4.2. Common Antioxidants Used in Dietary Supplementation

#### 4.2.1. Vitamin Supplements

Vitamin E: Recognized primarily as α-tocopherol, vitamin E, stands out as a robust antioxidant that effectively disrupts chain reactions [74]. Being lipid-soluble, it predominantly resides within cellular membranes, playing a paramount role as a primary chain-breaking antioxidant. By quenching free hydroxyl radicals and superoxide anions, it effectively curtails the lipid peroxidation process initiated by ROS at the membrane level [75]. A consistent relationship has been observed between seminal plasma’s vitamin E concentrations and the motility rate of spermatozoa [74]. Additionally, infertile men tend to exhibit reduced vitamin E levels in their semen [76].

Clinical studies have underscored its potential in enhancing fertilization rates by curbing lipid peroxidation [77]. Kodama et al. observed that a regimen inclusive of vitamin E led to augmented sperm concentration and diminished DNA damage in infertile patients, although the motility and morphology remained largely unchanged [78]. Another study highlighted the synergistic benefits of vitamin E and selenium, which collectively improved sperm motility and reduced the incidence of defective spermatozoa [79,80].

The antioxidant prowess of vitamin E is further accentuated by its ability to mitigate oxidative damage, subsequently elevating fertilization rates [81]. This is corroborated by extensive research emphasizing the role of antioxidants, including vitamin E, in neutralizing oxidative stress and free radicals [82]. Greco et al.’s study is particularly illuminating, revealing how a combined therapeutic approach with vitamins E and C significantly ameliorated sperm DNA fragmentation issues, leading to enhanced clinical pregnancy and implantation outcomes [83].

Vitamin E’s direct neutralization of free radicals, especially when paired with CoQ10, offers a protective shield against lipid membrane peroxidative damage [84]. Moslemi et al.’s research further substantiates this, showcasing how vitamin E supplementation can markedly diminish lipid peroxidation in seminal plasma, bolster sperm motility, and enhance pregnancy outcomes [85]. However, it is imperative to note that while a majority of studies vouch for the benefits of vitamin E on semen parameters, a meta-analysis did indicate some studies showing minimal or no impact [86]. Furthermore, its presence prevents peroxidation cascade and enhances other antioxidant compounds [87]. Research by Rengaraj and Hong have demonstrated how a deficiency in vitamin E may lead to abnormalities in spermatogenesis, and although supplement doses have not proven any significant increases in quality sperm production, they still show positive effects on testis health and sperm function [88]. Notably, research conducted with men experiencing infertility issues has demonstrated its success at decreasing ROS production [89].

Vitamin C: Similar to vitamin E, vitamin C, also known as ascorbic acid, works as an antioxidant protecting cells against ROS. This primary naturally occurring water-soluble antioxidant is an essential dietary nutrient. It has a pronounced capacity for scavenging ROS [90] and serves as a valuable electron donor, supplying electrons to free radicals like superoxides and hydroxyl radicals, thereby reducing their reactivity and potential damage [91]. This dual nature, acting as both an antioxidant and prooxidant, is contingent upon its concentration [92].

In semen, ascorbic acid plays a pivotal role in regulating oxidative stress [93]. Notably, it is found at a concentration 10 times greater in seminal plasma compared to blood serum [94]. This elevated concentration suggests its significant role in protecting DNA integrity, with increased dietary intake correlating to higher concentrations in seminal plasma [95]. Furthermore, vitamin C has the ability to neutralize specific radicals such as hydroxyl, superoxide, and hydrogen peroxide, offering protection against endogenous oxidative damage [96]. Seminal fluid analyses of infertile males diagnosed with asthenozoospermia revealed diminished levels of vitamin C and elevated ROS levels compared to their fertile counterparts [97].

Vitamin C also acts as a cofactor for key enzymes, aiding in the metabolism of folic acid, tyrosine, and tryptophan [98], and interacts with glutathione to maintain the reduced form of tocopherol [99]. Research has highlighted its protective role against oxidative damage in the Leydig cells, sperm, and steroid cells within the sperm chamber [100]. A synergistic combination of vitamins C and E has been observed to shield spermatozoa from peroxidative damage and DNA fragmentation [101].

Cyrus et al. [102] conducted research which suggested that while taking supplemental vitamin C did not increase sperm count it did help increase motility and morphology, although its exact benefits remain uncertain; in order to assess its role in mitigating male infertility more rigorous clinical research would need to take place [103]. Gerco et al. [83] conducted an interventional study aimed at male infertility. Participants assigned to the intervention group received 1 g each of vitamin E and C daily for two months; post-study analysis demonstrated a substantial decline in DNA damage. However, seminal traits such as motility and concentration did not experience significant improvements with the combined vitamin E/C regimen. It was highlighted that antioxidant blend could play an integral part in mitigating sperm DNA damage during assisted reproductive techniques such as intracytoplasmic sperm injection (ICSI) and IVF [83].

Vitamin B12, commonly referred to as cobalamin, plays an essential role in metabolism of fats and proteins [104]. Vitamin B12 also has been proven to enhance sperm quality by decreasing DNA fragmentation while simultaneously increasing count and motility [105]. Furthermore, its use as an antidote for cryodamage protection during assisted reproductive technology (ART) freeze–thaw processes has been explored extensively [106]. When added into cryopreservation medium, it has shown improved survival and motility, as well as a reduction in DNA fragmentation [106]. Overall, there is compelling evidence suggesting that vitamin B12 combined with other antioxidants shows promise in addressing subfertility issues; however, its exact mechanisms remain to be fully comprehended.

Vitamin D plays a pivotal role in various physiological processes, including the regulation of fertility in both males and females [107,108]. It is intricately linked to the quality of spermatozoa and is essential for maintaining sperm motility [109]. Reduced levels of vitamin D in men have been associated with diminished reproductive capabilities [110]. Vitamin D is crucial for reproductive health. A deficiency in vitamin D has been shown to decrease the success rates of assisted reproductive technology procedures [111,112,113]. Research by Islamian et al. delved into the combined effects of vitamin D supplementation and docosahexaenoic acid on oxidative stress markers in the semen of men diagnosed with asthenospermia. Their findings highlighted that a combined therapy of docosahexaenoic acid (a fatty acid supplement) and vitamin D led to a significant reduction in oxidative stress levels within the seminal plasma, thereby potentially improving reproductive outcomes [113].

Folic acid (vitamin B9) plays a central role in nucleic acid synthesis and amino acid metabolism, as well as possessing antioxidant properties that neutralize reactive oxygen species, making it an attractive treatment option for male infertility [114]. Folic acid works synergistically with zinc, yet insufficient evidence supports treating infertile males solely with folic acid monotherapy. While studies have examined this relationship, one such investigation revealed an elevation in peripheral blood inhibin B levels [115], an indicator of successful spermatogenesis as it measures Sertoli cell health [62]. Raigani et al. [116] found that neither folic acid or zinc, taken individually or combined, significantly improved sperm concentration, motility, or morphology; however, Irani et al.’s comprehensive review indicated otherwise: combined folate and zinc supplementation provided superior effects compared to placebo on improving sperm concentration, motility, and morphology, as well as increasing serum folate levels; thus suggesting that taking folate alone may not be more efficacious [117].

#### 4.2.2. Zinc

Zinc can be found in higher concentrations in human sperm than blood, reflecting its production from the prostate gland [118]. As a vital trace element, zinc has many biological functions, playing a pivotal role in the metabolic pathways of RNA and DNA, signal transmission, and gene expression [119]. Furthermore, zinc finger proteins play a pivotal role in regulating the genetic expression of steroid hormone receptors [120], emphasizing its multifunctional nature in cellular processes. Its antioxidant capabilities are believed to arise from its capacity to reduce the generation of hydrogen peroxide and hydroxyl radicals by counteracting redox-active transition metals, such as iron and copper [121].

Zinc is essential in many physiological processes including DNA repair, cell cycle progression, and apoptosis [122]. Its presence in seminal plasma serves as an antioxidant and antibacterial agent, providing protection against the buildup of heavy metals [123]. Zinc is essential to normal testicular development and spermatogenesis. Zinc plays an essential role during meiosis during spermatogenesis as well as various stages of gametogenesis [124]. The administration of the vitamin zinc has been shown to be particularly effective in improving sperm morphology and DNA integrity in individuals with prostate problems, underscoring its crucial role in DNA compaction processes [125,126]. Elevated concentrations of zinc in seminal fluid can, however, negatively impact the acrosome response generated by spermatozoa-zona pellucida binding in males with normal sperm parameters [127].

Furthermore, zinc plays a crucial role in the production, storage, secretion, and function of numerous enzymes [128]. These enzymes, under the influence of zinc, are instrumental in meiosis during spermatogenesis and various stages of gametogenesis. Its involvement in these processes underscores its significance in ensuring the proper progression of spermatogenesis, from the early stages of germ cell development to the maturation of spermatozoa [124,128]. Finally, due to its effect on lipid fluidity it impacts cell membrane integrity as well as sperm chromatin integrity [129]. Zinc levels in seminal plasma display a specific pattern: they are highest among normozoospermics, then asthenoteratozoospermics, then oligoasthenoteratozoospermics, and finally, are lowest among azoospermics [130]. Research by Colagar et al. has highlighted how an inadequate zinc intake may contribute to impaired sperm quality and male infertility, leading them to advocate for measuring zinc levels during infertility investigations [131]. Alsalman et al. conducted a randomized clinical trial that compared zinc supplementation on seminal plasma between fertile and infertile men. Their findings demonstrated that among infertile men, zinc supplementation enhanced sperm quality through the elevated activity of zinc-dependent enzymes that improved motility [132]. Ebisch et al. [133] studied the effects of supplementing men with 66 mg of zinc and 5 mg of folic acid over 26 weeks, finding an increase in sperm count while other attributes remained unaltered; key indicators like motility, serum sperm concentration levels, inhibin B levels, and zinc levels showed positive correlations when compared with their baseline values.

Other improvements included stimulation of spermatogenesis, advancement of sex organ maturation and activation of 5-reductase [132]. Hadwan et al. [134] reported on changes in sperm characteristics among oligoasthenoteratozoospermia (OAT) diagnosed males following zinc supplementation. Their study included 60 fertile and 60 infertile male participants, who received two capsules of zinc sulfate (220 mg each daily for three months). Notably, the outcomes showed improvements in sperm parameters such as total normal sperm count, volume, and percentage of progressive motility. In order to further explore mechanisms behind these improvements, this research noted zinc binding sites present in seminal plasma. Hadwan’s research further illustrated that fertile men possessed higher proportions of high-molecular-weight ligands than their infertile counterparts, and treated men with OAT saw an increase in high-molecular-weight ligands and normalization of low-molecular-weight ligands [135]. Zinc supplementation has been demonstrated to improve in vitro sperm motility and capacitation; however, no conclusive proof exists for its efficacy in clinical settings [136]. More extensive research should be conducted to ascertain both its benefits and optimal dosage in treating male infertility.

#### 4.2.3. Selenium

Selenium (Se) is an integral component of several types of proteins known as selenoproteins, playing critical roles in antioxidant defense, redox state modulation and cancer prevention [137]. Se plays a pivotal role in various physiological processes, particularly through its incorporation into selenoproteins. These selenoproteins, including the notable glutathione peroxidase, are central to selenium’s antioxidative properties [138]. They actively combat oxidative stress by neutralizing harmful reactive oxygen species ROS, thereby protecting cellular structures from oxidative damage [139]. This antioxidative mechanism is crucial in preventing the onset of various diseases, including certain cancers [140]. Moreover, selenium’s role extends beyond antioxidative defense. It is instrumental in modulating the redox state of cells, ensuring a balance between oxidation and reduction processes [138]. This balance is vital for various cellular functions, including DNA synthesis and repair, protein synthesis, and enzymatic reactions [141]. Additionally, selenium’s involvement in cancer prevention is believed to be linked to its ability to maintain genomic stability, reduce DNA damage, and modulate immune responses [140]. In the context of reproductive health, selenium’s antioxidative properties are particularly significant. It safeguards the sperm from oxidative damage, ensuring its viability and motility, which are essential for successful fertilization [67]. Furthermore, selenium’s role in DNA integrity is crucial for the proper development and maturation of spermatozoa [67].

Se is an essential cofactor in antioxidative enzymes, providing essential support in neutralizing ROS while simultaneously preventing their formation during crucial processes like spermatogenesis, mitochondrial activity, and capacitation [142]. Glutathione peroxidase stands out among selenoproteins for male reproductive health due to its multiple redox processes and presence within mitochondrial membranes of spermatozoa as a regulatory mechanism, mitigating ROS production during motility [143]. Selenium is essential to both testosterone synthesis and sperm development [144]. Humans possess over 25 selenoproteins that support structural integrity of sperm. Selenoprotein P (SePP), another notable selenoprotein, plays an essential role in normal spermatogenesis. This selenoprotein is crucial for maintaining the structural integrity of spermatozoa within the testes. Deficiencies in SePP have been linked to abnormalities in sperm tails, including structural disruptions in mitochondrial membranes and microtubules, as well as alterations in the central and main components of the spermatozoon [145]. While supplementation can address deficiencies stemming from diet, SePP is indispensable for maintaining selenium balance and ensuring the proper development of sperm [145]. Moreover, SePP is not only abundant in the testes but also in seminal fluid, suggesting its pivotal protective role during the various stages of sperm storage and transit through the reproductive tract to fertilization [146].

Selenium’s influence on testosterone production and secretion is also noteworthy. Rodent studies have shown that when selenium intake is limited, it is preferentially directed to the testes [147,148]. This prioritization underscores selenium’s importance in testicular function. Additionally, research indicates that selenium supplementation can elevate testosterone levels within the testes, hinting at an interplay between selenium and hormones involved in spermatogenesis [147,148]. In conditions of selenium deficiency, hormonal stimulations, such as those from luteinizing hormone-releasing hormone (LHRH) or chorionic gonadotropin (hCG), resulted in only modest increases in serum testosterone levels compared to selenium-adequate conditions. This suggests that a lack of selenium might modify the receptors of the luteinizing hormone (LH) on Leydig cells, subsequently influencing testosterone secretion [149]. In essence, selenium, through its selenoproteins and interactions with hormonal pathways, plays a pivotal role in both sperm and testosterone production.

Found abundantly both in testis and seminal fluid, SePP plays an integral part in safeguarding sperm during its storage, transit through reproductive tract, transformations, and interaction with an oocyte [146]. Studies have demonstrated a correlation between seminal plasma SePP concentrations and both concentration and viability [146]. Mistry et al. [150] reported daily Se supplementation was found to enhance various sperm parameters such as count, concentration, morphology, and motility. Safarinejad et al. conducted an experimental 30 week study of selenium and N-acetyl-cysteine on 468 men diagnosed with idiopathic OAT over 30 weeks, showing increased testosterone and inhibin B levels while decreasing levels of FSH; remarkably, all semen parameters improved, with selenium amplifying these positive results on semen quality [151].

Furthermore, findings by Taleby et al. [152] highlighted that the increased consumption of selenium through diet was associated with improved motility as well as an increase in ejaculate volume. But when it comes to selenium, striking the appropriate balance is key for its safety and wellbeing. Sustaining appropriate levels in both blood and seminal plasma is important to avoid toxicity; mismanaged supplementation could upset ROS balance necessary for processes like motility and acrosomal reaction, potentially restricting male reproductive potential [153]. Studies involving fertile men have highlighted how too much Se can lead to changes in sperm morphology as well as decreased motility [154].

Recent research highlights potential drawbacks associated with excessive antioxidant supplementation. Sengupta et al., 2022, warned of an excess of antioxidant supplementation interfering with vital oxidation systems and negatively affecting fertility [155]. Likewise, Sadeghi et al., 2022, observed that excessive antioxidant supplementation could induce reductive stress, leading to poorer sperm quality that reduces intended benefits [156]. Optimized serum Se levels have been associated with superior sperm morphology; exceeding these levels, however, can compromise it. With so much emphasis placed on using megadoses of Se as an antioxidant supplement recently [157], clinical research should continue in order to ascertain an ideal dosage [75].

#### 4.2.4. Coenzyme Q10

Coenzyme Q10 (CoQ10) plays a central role in the synthesis of ATP, the primary energy currency for cells, as well as acting as an effective antioxidant [158]. It is preset in mitochondrial respiratory chains and servs to control reactive oxygen species production via electron transport cycling processes such as oxidative phosphorylation; its presence helps protect membrane damage caused by peroxidation [159]. Sperm cells utilize electron transport cycle mechanisms primarily provided by mitochondria using oxidative phosphorylation; CoQ10 neutralizes reactive oxygen species produced during electron transport cycles [160].

Sperm mitochondria exhibit a notable abundance of this substance at elevated levels, which is intricately associated with cellular respiration and serves as a fundamental component in the generation of energy [161]. This contribution provides a rationale for its use as a chemical with promotility and antioxidant properties. In addition, CoQ10 has been shown to effectively suppress the production of superoxide, thus providing a safeguard against oxidative stress-induced impairment of sperm function. Previous research has shown a notable inverse relationship between levels of CoQ10 and hydrogen peroxide. Additionally, a linear association has been seen between CoQ10 levels in seminal plasma and both sperm count and motility [162].

Notably, infertile men exhibit reduced levels of CoQ10 [158]. CoQ10 levels have a direct correlation with compromised sperm parameters, including motility. Studies have demonstrated that CoQ10 can increase both the count and motility of sperm, offering potential solutions to infertile men [163,164]. Balercia et al. studied the effect of CoQ10 on sperm motility among infertile men in a randomized, double-blind trial involving 60 males with idiopathic OAT. Their administration resulted in higher CoQ10 concentrations in their ejaculate after six months, leading to enhanced motility. Their results also demonstrated six natural pregnancies among CoQ10 participants as compared to three [165].

Nadjarzadeh et al. conducted a double-blind, randomized clinical study to explore the role and potential benefits of CoQ10 supplementation in improving seminal parameters in males with OAT, with their findings emphasizing increased OS levels among OAT males—something which negatively impacted semen parameters as well as functional sperm production and motility morphology. CoQ10 levels were directly correlated with both motility morphology and motility via seminal plasma analyses, and the research demonstrated how three months of supplementation led to decreased OS levels while simultaneously increasing antioxidant enzyme activity [166].

Garca-Daz et al. provided further evidence of CoQ10’s beneficial properties by showing that supplementation for three months significantly enhanced sperm concentration, overall motility, and progressive motility [167]. Safarinejad et al. [168], who conducted a subsequent randomized controlled trial involving 228 infertile men with compromised sperm parameters, reported improved density, morphology, and motility after 26 weeks of CoQ10 treatment in their intervention group. Thakur et al. [169] found a positive relationship between seminal concentrations of CoQ10 and key semen attributes such as concentration, motility, and morphology with increased overall antioxidant capacity due to this correlation. Coenzyme Q10 remains to be given its proper dosage; however, with all of the challenges inherent to treating male infertility idiopathic, CoQ10 stands out as one of the most promising therapeutic agents [158].

Contrasting meta-analyses suggested otherwise. While certain studies did demonstrate improved sperm parameters with supplementing CoQ10, it did not significantly increase live birth or pregnancy rates in infertile men [159]. Still, this meta-analysis acknowledged CoQ10 as beneficial in terms of improving motility and concentration parameters in particular sperm samples [22,170].

#### 4.2.5. L-Carnitine

Carnitines, namely L-carnitine (LC) and L-acetyl carnitine (LAC), are water-soluble antioxidants that play a crucial role in sperm metabolism by providing energy for essential processes such as sperm motility [171]. LC is an endogenous metabolite of 3-aminobutyric acid in humans, and plays an essential role in intermediate metabolism—specifically in the production of acyl carnitine esters [172]. The findings of previous research indicate that the inclusion of carnitines in the culture conditions used for in vitro investigations of sperm resulted in increased motility and viability when compared to control groups [173]. The antioxidant properties of the subject are shown by their ability to scavenge superoxide anions and hydrogen peroxide radicals, thus preventing lipid peroxidation [174]. The study found a significant decrease in carnitine concentrations in semen samples obtained from infertile men diagnosed with oligoasthenoteratozoospermia [175].

Epididymal concentrations of LC exceed those found in serum by approximately 2000 times [176,177], likely owing to its consistent secretion by glandular epididymis tissue [172]. Additionally, elevated concentrations are positively correlated with improved sperm mobility [172]. In terms of an increase in L-acetyl levels within semen [177]. However, studies exploring LC as an antioxidant are few and far between.

Lenzi et al. conducted a randomized, controlled clinical trial investigating its therapeutic benefits in treating male infertility using OAT diagnosis alone. Their trial divided 60 male OAT-diagnosed infertile participants into intervention and control groups, with only the intervention group receiving daily dosages of 2 g LC and 1 g L-acetyl carnitine (LAC). Their findings demonstrated an increasing correlation between daily dosages of both supplements and improved sperm motility—which was even stronger among those whose initial motility was suboptimal [178].

Balercia et al. [179] provided further evidence of the efficacy of LC and LAC through their double-blind, randomized study with 60 males who suffered from OAT due to idiopathic causes. A six-month regimen consisting of both treatments led to improvements in both sperm motility and total oxygen radical scavenging capacity (TOSC), with nine participants reporting successful pregnancies (five leading to healthy birth). Sigman et al.’s [180] study reported diverging results. There was no significant improvement in either sperm concentration or motility after treatment with either LAC or LC.

Garolla et al. [181] conducted an additional double-blind trial utilizing the combination of LC with phospholipid hydroperoxide glutathione peroxidase (PHGPX) for treating male OAT. A double-blind study of 30 men employed two distinct treatment protocols; one group received a placebo for three months followed by 2 g of LC twice daily treatment over another three month period, ultimately showing improved sperm motility among subjects, particularly in those who maintained standard levels of PHGPX post-treatment. The outcome showed improvement across subjects; especially noticeable were those with standard levels post treatment of both.

#### 4.2.6. L-Arginine

L-arginine plays a pivotal role in various cellular processes, including the regulation of host defense mechanisms and cellular immune responses [182]. It is integral to the production of nitric oxide (NO), a transient-free radical synthesized in several mammalian cell types via nitric oxide synthases (NOS) [183]. These enzymes catalyze the conversion of L-arginine to L-citrulline and NO [184]. NO has been identified as a key modulator in sperm capacitation, with empirical data suggesting that low concentrations of NO can enhance human sperm capacitation [185]. The activation of NO production is also linked to the augmentation of tyrosine phosphorylation in sperm cell proteins, a crucial step towards sperm capacitation [186].

L-arginine serves as a protective agent against the peroxidation of membrane lipids, which is essential for maintaining sperm quality [62]. A deficiency in L-arginine can disrupt sperm metabolism, leading to reduced motility and impaired spermatogenesis [182]. In vitro studies have shown that L-arginine can enhance sperm motility, especially in samples with poor motility [187]. Furthermore, L-arginine’s ability to enhance NO production suggests its protective role for spermatozoa against lipid peroxidation. Clinical investigations have demonstrated the potential of L-arginine supplementation in improving sperm motility and overall sperm quality in infertile males [188]. Additionally, its administration has been linked to a decrease in lipid peroxidation levels [189] and an increase in both sperm count and motility [190]. In a controlled trial, L-arginine supplementation resulted in significant improvements in sperm quality parameters, such as volume, concentration, motility, vitality, and morphology, without any adverse effects [191].

#### 4.2.7. Lycopene

Lycopene, a carotenoid abundant in fruits and vegetables, plays a vital role in the human redox defense system with its potent ROS neutralizing properties [192]. Within the male reproductive framework, significant concentrations of lycopene are found in the testes and seminal plasma. Notably, reduced levels are observed in males facing infertility, underscoring its importance in reproductive health [193]. While its antioxidant properties are well-recognized, lycopene’s presence in the testes also hints at its contribution to spermatogenesis [194]. Its potential in safeguarding sperm integrity suggests a promising avenue for enhancing fertility. Further studies are essential to fully understand its impact on reproductive health.

#### 4.2.8. Inositols

Myoinositol (MI) is the primary inositol found in nature, and its transport into cells is facilitated by sodium/MI cotransport protein produced in response to changes in osmotic pressure. The existence of this protein is especially essential because MI cannot pass through tight junctions at testicular level alone [195], leading to higher concentrations within seminiferous tubules than in seminal plasma [196]. MI plays an essential role in regulating oxidative metabolism and ATP production within spermatozoa by modulating intracellular Ca^2+^ levels in their mitochondria, supporting mitochondrial activity that boosts processes such as capacitation, the acrosome response, and motility [62]. Such effects have been observed among patients with suboptimal sperm parameters, where MI administration resulted in improved motility [197]. Furthermore, research by Governini et al. revealed that MI treatment significantly increases motility [198]. MI is essential in several post-ejaculation processes of spermatozoa, such as capacitation and the acrosome response [199], as well as interactions between sperm and oocytes [200].

Furthermore, research by Calogero et al. [199] highlights its safety by showing its ability to significantly normalize serum levels of gonadotropins and inhibin B. Studies conducted in vitro have demonstrated that increasing mitochondrial ATP production with MI [17,30,31] can provide protection from irregular sperm morphology while simultaneously improving motility over time and across sperm pools [198,201,202]. Overall, MI is vital to numerous processes essential for the production and fertilization of sperm. Through participation in mitochondrial reactions, it assists in controlling oxidative stress levels in sperm cells while restoring hormonal equilibrium among men with infertility issues.

#### 4.2.9. Alpha-Lipoic Acid

Alpha-lipoic acid (ALA) plays a central role in ATP synthesis, an essential process for male reproductive success. More specifically, it serves as an essential cofactor in mitochondria for both pyruvate dehydrogenase and alpha-ketoglutarate dehydrogenase [203]. Once inside cells and tissues, ALA transforms into dihydrolipoic acid (DHLA), an even stronger antioxidant with the power to neutralize reactive oxygen species while simultaneously chelating transition metals, thereby protecting membrane lipid peroxidation or protein damage [204,205]. Haghighian et al. conducted an intensive randomized, triple-blind, placebo-controlled clinical trial comparing the effects of taking an ALA supplement for three months against those seen when taking a placebo pill over three months; those taking the latter showed substantial improvements in terms of concentration, count, and motility [206]. Taherian et al. investigated the effects of ALA supplementation on intracellular oxidative stress levels and fragmented sperm DNA fragmentation rates among infertile men and found that in vitro supplementation significantly reduced both measures through the reduced production of reactive oxygen species [207]. Di Tucci et al. conducted an exhaustive investigation of ALA’s impact on fertility, discovering that its administration significantly enhanced several semen parameters, including motility, morphology, and count [208]. Their study also demonstrated its potential antioxidant protection against ROS damage [209]. Varicocele, a condition linked to male infertility, has also been the focus of ALA research. Following varicocele surgery, supplementation was investigated as a potential fertility enhancer by Abbasi et al.’s double-blind placebo-controlled trial; their research concluded that post-surgery ALA treatment led to superior sperm quality compared with controls 80 days post-surgery [210]. As part of ART, ALA was investigated to evaluate its potential as a cryoprotective agent during the freeze–thaw process for sperm. Due to risks related to oxidative stress and cryodamage during cryopreservation, Asa et al. found an optimal concentration of ALA to provide protection from ROS production and cryodamage; furthermore, their findings demonstrated an increase in motility, viability, and reduced DNA damage and apoptosis [211]. Overall, ALA has proved itself to be a highly effective means of improving male fertility through various means, including increasing sperm parameters or offering protection during cryopreservation processes.

#### 4.2.10. Considerations for the Dosage, Duration, and Potential Side Effects of Antioxidant Supplementation

Consultation with Healthcare Professionals: Before starting any antioxidant supplements for male reproductive health, it is imperative to consult a healthcare expert or reproductive specialist. These professionals can assess individual needs, consider any pre-existing medical conditions or medications, and offer tailored advice [64].

Dosage: It is worth underscoring that prior randomized controlled trials (RCTs) and meta-analyses did not examine the best dose and timing of medicine. To date, there is a lack of precise definition on the specific doses and regimens [82]. The ideal dosage of antioxidants varies based on the specific antioxidant and its formulation. Always follow the dosage guidelines provided on supplement labels or as recommended by a healthcare specialist. Dosages can differ based on factors like age, overall health, and specific fertility concerns [212]. The commonly administered dosages include [72]:

- Vitamin C: 500–1000 mg;

- Vitamin E: 400 mg;

- Zinc: 25–400 mg;

- Carnitines (L-carnitine (LC) or L-acetyl-carnitine (LAC)): 500–1000 mg;

- CoQ10: 100–300 mg;

- Selenium (Se): 200 mg;

- Folic acid: 0.5 mg;

- Lycopene: 6–8 mg.

Duration: Antioxidant supplements often require consistent administration over a certain period to show noticeable effects on sperm parameters. Improvements in sperm quality might take several months, given that the sperm regeneration cycle is approximately 74 days. It is vital to maintain consistency in taking the supplements as per guidelines and to be patient, allowing sufficient time for the supplements to work [213].

Potential Side Effects: A recent research has shown that an overconsumption of antioxidants may disrupt the balance between oxidation and reduction, leading to a state of reductive stress. This condition has been found to have a similar negative impact on the quality of sperm as OS [214]. The “antioxidant paradox” is a term introduced by Halliwell et al., highlighting a potential downside to the excessive use of antioxidant regimens [215]. While it is commonly believed that oxidative stress is harmful and can be countered by replenishing the body’s defense systems with reducing agents, this perspective might be overly simplistic and even risky. Overexposure to these reducing agents can lead to what’s termed as reductive stress, which bears similarities to oxidative stress [216].

Research has shown that the excessive intake of antioxidants can have adverse effects on the cardiovascular and neural systems, leading to conditions like cardiomyopathy and disruptions in the brain’s microvasculature [216,217,218,219,220]. Such stress can also hinder endothelial cell growth and compromise mitochondrial function [221,222]. In the realm of male reproductive health, reductive stress implies that the overuse of readily available reducing agents might upset the balance of redox reactions, negatively impacting fertility [214]. Elevated levels of these reducing agents have been linked to a range of issues, from diminished oxidative capacity and tissue damage to an unexpected surge in ROS [215].

#### 4.2.11. Exploring the Synergy of Multiple Antioxidants

Recent research has explored the use of multiple antioxidants in treating male infertility [223,224,225]. Bish et al. emphasize the synergy created when multiple antioxidants are combined together by emphasizing their collective roles in neutralizing reactive oxygen species (ROS) produced through enzyme activity, while mitigating production through natural means such as diet. They recommend the consumption of food that are rich in zinc and selenium [226]. Santoro et al. investigated the effects of supplementing with an antioxidant blend primarily composed of MI, studying its impacts both in vitro and in vivo. Their findings suggested that antioxidant regimens could possibly enhance semen preparation for IVF, with no reported risks tied to orally consuming this nutraceutical blend, particularly when targeting the quality of OAT sperm [227]. Scaruffi et al. also demonstrated this by administering various antioxidants prior to semen collection, yielding improved fertilization rates. Both studies demonstrated how further research needs to be conducted to understand outcomes completely [227,228]. Yet a recent multicenter, double-blind, randomized, placebo-controlled trial provided contradictory findings. It concluded that providing combined antioxidant treatment to male partners did not increase in vivo pregnancy rates or live birth rates or improve semen metrics or DNA integrity in infertile men [229]. Vegetables, being rich in antioxidants, play a pivotal role in this discussion. There is evidence pointing to diet as an integral factor in improving sperm quality; thus, it has often been recommended that men opt for plant-rich diets such as vegetarianism, DASH, or Mediterranean regimens either alone or coupled with antioxidant supplementation [230,231]. However, the exact efficacy of combined antioxidant regimens remains somewhat indeterminate due to factors such as non-homogeneous demographics of clinical trial groups and lack of rigorously conducted, placebo-controlled double-blind studies. A recent review suggested that current antioxidant treatments might not truly boost sperm functionality while their significant costs may dissuade individuals from seeking them long term [232].

## 5. Antioxidants in Clinical Perspective: A Therapeutic Beacon

Oxidative stress, due to ROS production, adversely affects sperm function and DNA integrity, with higher ROS levels correlating negatively with assisted reproductive technology outcomes [233,234]. Antioxidants have emerged as a promising therapeutic avenue for addressing male infertility. Notable antioxidants like vitamin C, CoQ10, L-carnitine, and glutathione have shown potential benefits in this context. However, the current scientific consensus on their efficacy is not definitive due to factors like the underreporting of randomization, high patient turnover, and small sample sizes in studies [235].

Idiopathic and Unexplained Male Infertility: The terms unexplained male infertility (UMI) and idiopathic male infertility (IMI) are frequently used to describe conditions where the root causes of male infertility remain elusive. UMI is characterized by the inability to conceive despite normal semen characteristics, while IMI presents with semen abnormalities of unknown origin. The prevalence rates of UMI and IMI range between 6% and 27% and 30% and 58%, respectively [236,237]. Oxidative stress is believed to significantly influence the pathogenesis of idiopathic infertility, being present in approximately 30–40% of UMI patients and in up to 80% of IMI patients [28,238,239].

Antioxidant Treatment in UMI and IMI: Antioxidants have been frequently employed in treating individuals diagnosed with UMI or IMI. The findings from our study suggest that antioxidant use in males diagnosed with IMI and UMI led to notable improvements in semen parameters and sperm function, as documented in methodologically rigorous studies. However, a significant number of low-quality studies reported similar gains, emphasizing the need for more trials to achieve statistical significance. A comprehensive analysis of 32 trials investigating the effects of antioxidant treatment on IMI patients showed a marked improvement in semen characteristics, especially sperm motility [240]. Limited studies have assessed the efficacy of antioxidant therapy in UMI patients. One such study involving 29 UMI-diagnosed individuals undergoing a 3-month antioxidant regimen reported a significant increase in progressive motility (*p* = 0.002) and a decrease in sperm DNA fragmentation (SDF) levels (*p* = 0.03) and oxidative–reduction potential (ORP) levels (*p* = 0.02) post-treatment [241].

Another study by Greco et al. [83] involving 64 UMI patients with increased SDF levels found a significant reduction in SDF in the group treated with vitamins C and E for two months, although semen parameters remained unchanged.

Yet, there remains contradictory information regarding the efficacy of antioxidant treatment in addressing male infertility. A Cochrane systematic review and meta-analysis, which encompassed 34 RCTs involving 2876 couples using various antioxidant compounds, suggested a beneficial effect of antioxidants on live birth and pregnancy rates among subfertile couples undergoing ART cycles [242]. A more recent meta-analysis, which included 61 trials with 6264 infertile males, echoed these findings [243]. However, the Males, Antioxidants, and Infertility (MOXI) study revealed that antioxidant administration did not significantly improve semen parameters or DNA integrity compared to a placebo, particularly focusing on males diagnosed with male factor infertility. Moreover, no significant difference was observed in the cumulative live-birth rate at 6 months between the antioxidant and placebo groups (15% vs. 24%) [229]. It is crucial to note that these studies have significant limitations, including a high risk of bias due to inadequate reporting and high attrition rates, compromising the precision of the results [243]. The evidence available does not conclusively indicate which specific antioxidants or treatment regimens are most effective in enhancing sperm parameters and increasing pregnancy rates [243].

In conclusion, while the potential benefits of antioxidants in treating male infertility, particularly in cases of UMI and IMI, are evident, a judicious approach backed by rigorous research is essential. Proper patient identification, dosage optimization, and continuous monitoring are pivotal to harnessing the full benefits of antioxidant therapy.

Semen Parameters and Antioxidant Therapy: The quality of semen is a crucial determinant of male fertility. Any deviation from the standard parameters can have profound implications on the reproductive potential of an individual. Agarwal et al. embarked on a comprehensive study to evaluate semen quality in individuals who deviated from the typical range, shedding light on the intricate relationship between oxidative stress, semen quality, and the therapeutic potential of antioxidants [244].

Oxidative stress, caused by excessive ROS, negatively impacts sperm quality and function. While many studies suggest that antioxidants can improve these issues, Agarwal et al. found that only high-quality studies showed less pronounced benefits from antioxidant therapy [244]. This observation underscores the importance of study design and the potential biases that can influence research outcomes. The recent Cochrane analysis further substantiated these findings, revealing a similar trend [243]. Although there was a discernible improvement in traditional semen parameters over time, the reliability of these results was compromised due to significant heterogeneity among the included studies. Notably, antioxidant therapy was found to reduce sperm SDF levels compared to a placebo, highlighting its potential therapeutic benefit [244].

Varicocele and the Role of Antioxidants: Varicocele, characterized by the enlargement and dilation of the veins within the scrotum, stands as a prominent correctable factor in male infertility. The prevalence of this condition is significant, affecting approximately 40% of men with primary infertility and a staggering 80% of those with secondary infertility [245]. The association between varicocele and elevated oxidative stress levels has been well-established, with multiple studies confirming higher oxidative stress in infertile men with varicocele compared to their fertile counterparts and those with idiopathic infertility [246,247,248,249,250]. This observation has naturally led to the exploration of antioxidants as a potential therapeutic intervention for varicocele-induced infertility.

However, the gold standard in managing varicocele remains varicocelectomy, a surgical procedure aimed at rectifying the dilated veins. This intervention has consistently shown promise in improving semen parameters and increasing the chances of natural conception in a majority of patients [251,252].

The therapeutic potential of antioxidants in the context of varicocele has been the subject of numerous studies. However, a significant portion of these investigations lacks methodological rigor. While many have reported positive outcomes with antioxidant supplementation, the collective evidence does not robustly advocate for antioxidants as a primary treatment for varicocele [244]. Yet, the improvements observed with antioxidant therapy cannot be dismissed outright. They suggest a potential role for antioxidants as an adjunctive treatment, especially when combined with varicocelectomy [244]. A recent meta-analysis, which included six RCTs with 576 post-varicocelectomy patients, provides further insight into this potential synergy. Patients who received antioxidant supplementation post-surgery exhibited significant improvements in various semen parameters, including sperm concentration, overall motility, progressive motility, and normal morphology. However, these enhancements did not translate to a significant rise in pregnancy rates [253].

## 6. Conclusions

Oxidative stress disrupts the delicate equilibrium between ROS production and antioxidant defense mechanisms. This disruption can lead to irreparable oxidative damage to sperm cells, impairing their viability and function. Ultimately, this can result in significant male reproductive health impairments. Supplementing with exogenous antioxidants is one method to mitigate these harmful effects. It allows for scavenging excess ROS, restoring equilibrium, and protecting sperm cells from further oxidative damage.

While certain antioxidants like vitamin C, CoQ10, L-carnitine, and glutathione have shown potential benefits, the scientific consensus on their definitive efficacy remains inconclusive due to various challenges in research methodologies.

It is also crucial to note that while antioxidants can offer therapeutic benefits, there exists a potential for overconsumption, leading to reductive stress. This paradoxical effect underscores the importance of a balanced approach to antioxidant therapy.

Overall, these findings indicate that antioxidant therapy could be an invaluable addition to current male infertility treatments. Antioxidants, by decreasing oxidative stress and improving sperm quality, may enhance the likelihood of successful conception for couples facing infertility. However, it is crucial to approach antioxidant therapy judiciously, considering the variability in individual responses and the potential interactions with other treatments. Further research is essential to determine the optimal dosage, duration, and the most effective specific antioxidants.
